# From Ototoxicity to Otoprotection: Mechanism and Protective Strategies in Cisplatin Therapy

**DOI:** 10.3390/ph18101543

**Published:** 2025-10-14

**Authors:** Andreea Iațentiuc, Sebastian Romică Cozma, Otilia Elena Frăsinariu, Ingrith Crenguța Miron, Iustin Mihai Iațentiuc, Lucia Corina Dima-Cozma, Raluca Olariu, Anca Postolache, Ana-Maria Laura Buga, Alexandru Stingheriu, Edilene Boéchat, Oana Roxana Bitere-Popa

**Affiliations:** 1 Department of Mother and Child Medicine, University of Medicine and Pharmacy Grigore T. Popa, 700115 Iași, Romania; andreeacimpan@yahoo.com (A.I.); ingridmiron@gmail.com (I.C.M.); ancap93@gmail.com (A.P.); anamaria_bnz@yahoo.com (A.-M.L.B.); 2 Doctoral School, University of Medicine and Pharmacy Grigore T. Popa, 700115 Iasi, Romania; iatentiuc_iustin@yahoo.com (I.M.I.); alexandru.stingheriu@gmail.com (A.S.); 3 Surgery II Department, ENT Discipline, University of Medicine and Pharmacy Grigore T. Popa, 700115 Iasi, Romania; scozma2005@yahoo.com (S.R.C.); raluca_bcn@yahoo.com (R.O.); 4 Department of Medical Specialties I, University of Medicine and Pharmacy Grigore T. Popa, 700115 Iași, Romania; cdimacozma@yahoo.com; 5 Department of Audiology, Speech Pathology and Physiotherapy, Faculty of Human and Health Sciences, Pontifical University of São Paulo, São Paul 01246-904, Brazil; eboechat@pucsp.br; 6 Biomedical Sciences Department, Faculty of Medical Bioengineering, University of Medicine and Pharmacy Grigore T. Popa, 700115 Iasi, Romania; oana.bitere@gmail.com

**Keywords:** ototoxicity, otoprotection, cisplatin, multidisciplinary team

## Abstract

Although cisplatin plays a vital role in chemotherapy protocols, its impact on hearing should not be overlooked. The ototoxic effects of cisplatin can lead to hearing loss. Childhood hearing loss can significantly impact various aspects of development. Understanding the mechanism of cisplatin-induced ototoxicity is crucial due to its high level of complexity. The process involves multiple interconnected steps, ranging from cisplatin absorption to its interaction with the cellular antioxidant defense system, nuclear DNA, mitochondria, and the cytokine cascade. Each of these interactions contributes to the overall pathophysiology of ototoxicity and is closely interrelated. Based on these, various hypotheses and conclusions were outlined, and we tried to analyze them as broadly as possible. Knowledge of these mechanisms has given rise to promising avenues and otoprotection strategies to combat ototoxicity. Although there is only one drug approved by the FDA (Food and Drug Administration), there are numerous drugs that target the mechanisms presented, but that need more evidence to be able to be used safely. In addition to these, the role of the multidisciplinary team should not be neglected and protocols should be established for periodic follow-up of patients treated with cisplatin to prevent hearing loss. This narrative review aims to point out all the aspects presented, based on the analysis of the literature and the conclusions drawn over time. We have selected the articles of interest and analyzed the studies that have obtained promising results to bring an overview of how cisplatin acts at the cochlear level, what can be done to combat these mechanisms, what solutions exist now and how we can prevent hearing loss.

## 1. Introduction

Platinum-based compounds, particularly cisplatin, are essential agents in cancer therapy for both adults and children, as they effectively inhibit tumor cell proliferation, invasion, and metastasis [1,2].

Although it is very effective in treating solid malignancies, including CNS tumors, its therapeutic index is narrow, and cisplatin brings with it numerous adverse effects. Ototoxicity is one of the most well known and studied adverse effects. The complexity of the mechanisms of production, the impact on the quality of life (the appearance of hearing loss caused by ototoxicity), and the lack of therapies to combat it place emphasis on prevention [2,3].

Childhood hearing loss can significantly impact various aspects of development. Even mild hearing impairment may interfere with academic performance, social interaction, self-confidence, and overall well-being [3]. If left untreated, hearing loss affects cognition and mental health, making it a significant risk factor for dementia in adulthood. The quality of life is greatly affected, especially in the case of children, because hearing loss at an early age can affect language learning and understanding, the emotional sphere, the cognitive sphere, and social interactions, all of which contribute to numerous complications in the child’s adaptation and integration into society. The ototoxic effects of cisplatin are not only limited to hearing loss, but also to the appearance of tinnitus or vestibular toxicity that is very difficult to diagnose and evaluate in children [3,4,5]. To date, there is only one FDA-approved treatment for the prevention of cisplatin-induced ototoxicity, namely sodium thiosulfate (STS) [6,7]. Therefore, the development of therapies that slow down or permanently stop the development of hearing loss is of particular importance.

This narrative review aims to highlight the main mechanisms involved in cisplatin-induced ototoxicity, to bring therapeutic targets and potential otoprotective strategies to the forefront, and to emphasize the importance of monitoring this highly complex process. Emphasis is placed on interdisciplinary collaboration, and we advocate for increased awareness regarding the need for standardized international protocols to prevent hearing loss, especially in pediatric patients treated with cisplatin, where the social impact is considerable. In this context, we encourage clinical studies with the hope of identifying promising therapeutic targets to counteract the ototoxic process.

## 2. Cisplatin Ototoxic Mechanisms

### 2.1. Absorption of Cisplatin at Cochlear Level

Once in the systemic circulation, cisplatin is transported throughout the body, including the inner ear. Due to the process of passive diffusion, cisplatin reaches the cochlear level. It can cross the blood–cochlear barrier (protective layer that regulates the passage of substances to the cells at the cochlear level). When cisplatin reaches the cochlea, it accumulates mainly at the level of the outer hair cells (part of the Organ of Corti). These cells are very sensitive to cisplatin and the more drug accumulates in their level, the faster they are destroyed [8,9].

One of the pathways through which cisplatin enters the cochlea involves copper transporter 1 (CTR1). Within the cochlea, CTR1 is expressed in outer hair cells, inner hair cells, the stria vascularis, and spiral ganglion neurons, facilitating the uptake of the drug and ultimately contributing to cellular apoptosis [10]. It has been observed that the transtympanic introduction of copper sulfate protects against cisplatin-induced ototoxicity by decreasing the entry of the drug into the cochlea, influencing CTR1. This is explained by CTR1’s increased affinity for copper. The increase in the concentration of extracellular copper leads to competition with cisplatin for CTR1, thus reducing the absorption rate of cisplatin. However, the applicability of this strategy is questionable due to the toxicity of copper, and rigorous monitoring is needed to have long-term results [10,11]. The development of precise and targeted methods to block CTR1 at the cochlear level could protect patients from hearing loss without compromising the benefits of chemotherapy.

In addition to CTR1, the organic cation transporter (OCT) is also involved in the penetration of cisplatin at the cochlear level. There are three genes encoding organic cation transporters, OCT1 (SLC22A1), OCT2 (SLC22A2), and OCT3 (SLC22A3). These 3 isoforms have been detected, expressed mainly in the liver and kidneys [12,13]. The OCT2 isoform has also been observed in the Corti organ and in the vascular stria. The single-nucleotide polymorphism (SNP) in the OCT2 gene is believed to offer a protective effect against ototoxicity in pediatric patients. Inhibition of these transporters using cimetidine has been associated with a reduction in both nephrotoxicity and ototoxicity caused by cisplatin [12,13]. If the process of penetration of cisplatin into the cells of the inner ear could be inhibited, then the process of ototoxicity could be controlled.

### 2.2. Cochlear ROS Generation System and Antioxidant Defenses

Hearing loss is attributed to the excessive production of reactive oxygen species (ROS) within the cochlea. Some hypotheses suggest that this process could be mitigated using antioxidants, compounds that may serve as otoprotective agents. ROS are also known to trigger cochlear inflammation, prompting numerous studies that support the use of anti-inflammatory agents in the prevention of hearing loss. Animal studies have indicated that G protein-coupled receptors, such as the adenosine A1 receptor and cannabinoid receptor 2, may help prevent hearing loss by enhancing ROS clearance and suppressing ROS production, ultimately reducing inflammation [8,14].

For proper function, the cochlea depends on heightened metabolic activity in essential regions, including the vascular stria, spiral ligament, and spiral prominence. The intense metabolic activity favors the reaction between electrons in the mitochondrial respiratory chain with oxygen, resulting in the superoxide (O2^−^) form. Stimuli such as loud noise can increase the metabolic activity of the cochlea and, with it, also increase oxidative stress at the cochlear level. The intense metabolic activity of the cochlea can make it susceptible to hypoxic processes and ischemic lesions. Ototoxic drugs increase the production of ROS, either by stimulating the enzyme pathways that favor this process or by stimulating the antioxidant systems [8,15].

There are several mechanisms activated by cisplatin that contribute to the onset of ototoxicity. One of the mechanisms is based on the antioxidant model and involves the excessive formation of ROS in the cochlea, after exposure to cisplatin, a process followed by the reduction in antioxidant enzymes. In treatment with cisplatin, reduced activity of cochlear glutathione (GSH) and antioxidant enzymes was observed at the cochlear level. All this will eventually lead to the apoptosis of the hair cells, support cells, cells in the vascular stria, and the auditory nerve [14,15,16].

Another mechanism that generates ototoxicity involves adenine dinucleotide phosphate oxidase 3 (NOX3) which is involved in the massive generation of ROS in the cochlea after activation produced by cisplatin. Systemic administration of NOX3 inhibitors could prevent this process and reduce hearing loss [15,17,18].

**Figure 1:**
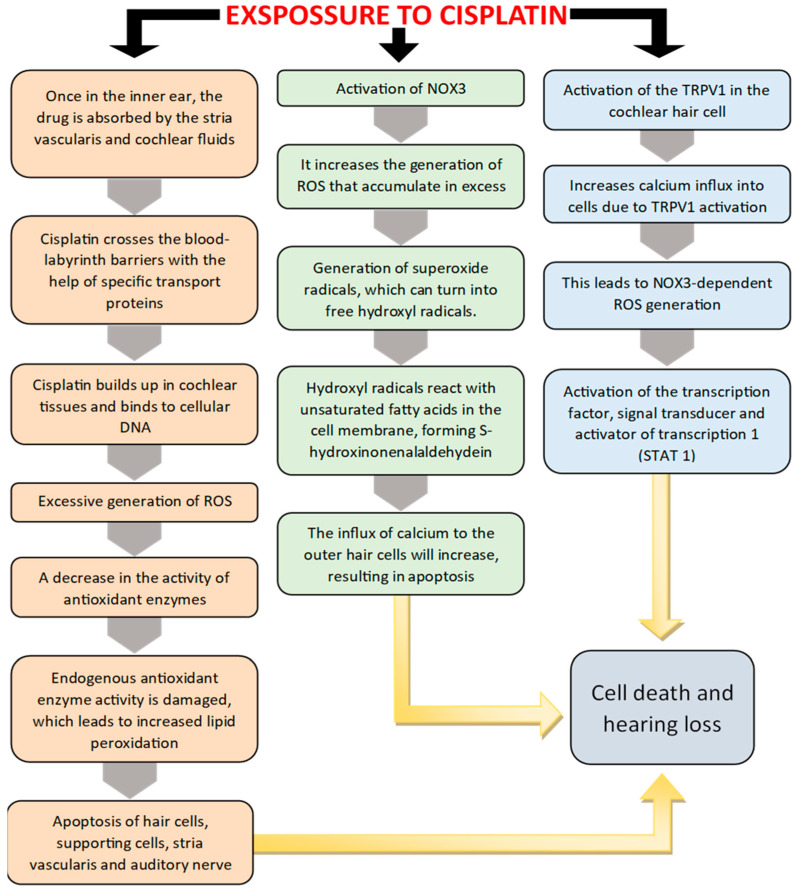
Cisplatin exposure and the mechanisms leading to hearing loss [7,8,14,15,16,18,19,20,21].

Another pathway that contributes to the occurrence of ototoxicity is through TRPV1 (Transient Receptor Potential Vanilloid 1) activation. Once activated, TRPV1 will lead to increased influx of calcium into cochlear cells, with excess ROS formation, which will lead to activation of the STAT1 (Signal Transducer and Activator of Transcription 1) pathway [19,20]. TRPV1 is a voltage-dependent cation channel, sensitive to stimuli such as high temperatures, low pH and various cellular stress factors. In the cochlea, this channel is expressed in sensory cells and plays a role in regulating calcium influx. In the context of cisplatin-induced ototoxicity, TRPV1 is abnormally activated, leading to the opening of the channel and massive calcium (Ca^2+^) influx into the cochlear cells. This increased calcium influx activates several intracellular signaling pathways, including enzymes such as protein kinases and phospholipases, which contribute to the amplification of oxidative stress. As a result, there is a significant increase in the level of ROS, which damage essential cellular structures such as membrane lipids, proteins, and DNA [19,21]. In the cochlea, hair cells are particularly vulnerable to these effects. ROS, in turn, activate the transcription factor STAT1, which promotes the expression of pro-apoptotic genes, leading to programmed cell death of auditory cells. Thus, TRPV1 activation in the presence of cisplatin contributes to the initiation of a pathological cascade that culminates in the apoptosis of hair cells and other supporting cells, leading to irreversible hearing loss. For this reason, the TRPV1 receptor is considered an important therapeutic target, and its inhibition represents a potential strategy for preventing the ototoxicity associated with cisplatin chemotherapy. (Figure 1) illustrates these pathways that lead to ototoxicity and hearing loss.

ROS can also be produced in the cochlea through the action of xanthine oxidase, an enzyme that transforms hypoxanthine (a byproduct of adenosine breakdown by adenosine deaminase) into uric acid. Blocking this enzyme with Allopurinol may help mitigate ototoxicity and nephrotoxicity, particularly when combined with a glutathione peroxidase (GSH-Px) mimetic [22,23].

The cochlea possesses an intrinsic antioxidant defense system composed of elements such as vitamins C and E, along with low molecular weight thiols like glutathione (GSH). Glutathione is found in the greatest abundance within the basal cells, the intermediate cells of the stria vascularis, and the cells of the spiral ligament [22,24]. Once penetrated the cell, cisplatin is transformed into a cisplatin monohydrate complex. This complex, in interaction with oxygen, generates superoxide (O2^−^). Reactive intermediates of cisplatin easily bind to reduced glutathione, converting it into its oxidized form (GSSG). Glutathione S-transferase, an enzyme responsible for detoxifying xenobiotics like cisplatin, plays a key role in this process. Higher expression of this enzyme in the cochlea may help neutralize cisplatin and lower the risk of ototoxic damage in this area [8,25].

**Figure 2:**
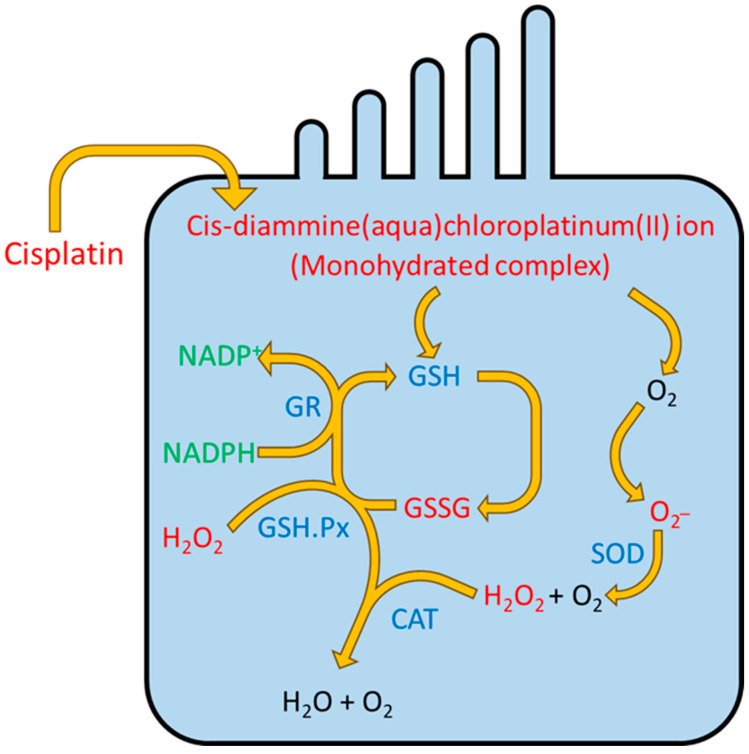
The cochlear antioxidant defense system.

The cochlea also contains several key antioxidant enzymes, including superoxide dismutase (SOD), glutathione peroxidase (GSH-Px), and catalase (CAT). SOD facilitates the conversion of superoxide anions (O_2_^−^) into hydrogen peroxide (H_2_O_2_) and oxygen (O_2_), while CAT further breaks down H_2_O_2_ into water and oxygen [8,18]. GSH-Px reduces hydrogen peroxide and other peroxides and plays a role in oxidizing reduced GSH into GSSG. Another important enzyme, glutathione reductase (GR), helps protect against oxidative stress by regenerating GSH from GSSG using reduced nicotinamide adenine dinucleotide phosphate (NADPH). Without this antioxidant defense system, cells in the cochlea would suffer significant damage due to lipid peroxidation, leading to elevated levels of harmful byproducts such as lipid peroxides, malondialdehyde, and 4-hydroxynonenal [8,25]. (Figure 2) shows the interaction of cisplatin with the antioxidant defense system.

There are studies that have observed that animals treated with cisplatin showed a dramatic decrease in GSH and a decrease in the activity of the antioxidant enzymes SOD, CAT, GSH-Px and GR. Multiple hypotheses have been proposed to explain this decline in antioxidant capacity [7,21]. The first hypothesis suggests that this reduction is caused by the covalent binding of cisplatin to sulfhydryl groups within key antioxidant enzymes, leading to their inactivation. Another hypothesis consists of the loss of metal cofactors (Cu, Se) vital for enzymatic activation. And the 3rd hypothesis would be represented by the excessive increase in ROS which leads to the depletion of antioxidant enzymes [8]. Elevated oxidative stress enhances lipid peroxidation of cellular membranes, impairs the activity of vital enzymes and membrane transporters, and interferes with the normal function of membrane ion channels. All of these will be responsible for cell apoptosis. Thus, the formation of ROS is undoubtedly a cause of cisplatin-induced ototoxicity, and inhibiting their production could stop the process of hearing loss [7,8,21].

Once inside the cell, cisplatin is converted into a monohydrated complex that generates superoxide (O_2_^−^). Reactive intermediates bind to reduced glutathione (GSH), forming its oxidized form (GSSG). Enzymes such as glutathione S-transferase, superoxide dismutase (SOD), catalase (CAT), glutathione peroxidase (GSH-Px), and glutathione reductase (GR) play key roles in detoxification and protection against oxidative stress. Together, these antioxidant defenses limit lipid peroxidation and prevent cochlear damage. GSH—antioxidant glutathione, GSSG—oxidized glutathione, GR—glutathione reductase, GSH.Px—glutathione peroxidase, SOD—superoxide dismutase, CAT—catalase, NADP^+^—nicotinamide adenine dinucleotide phosphate.

**Figure 3:**
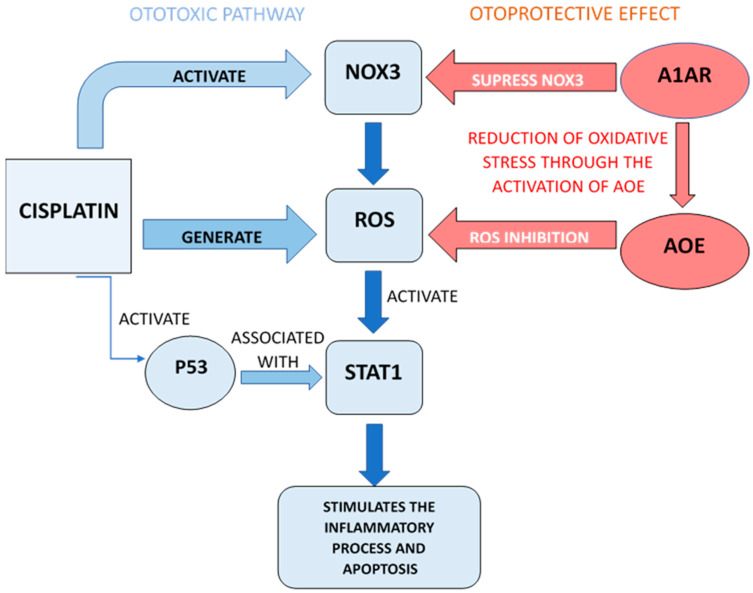
The otoprotective role of A1AR in reducing oxidative stress. NOX3—adenine dinucleotide phosphate oxidase 3, ROS—reactive oxygen species, STAT1—Signal transducer and activator of transcription 1, P53—tumor antigen p53, A1AR—adenosine A1 receptors, AOE—antioxidant enzymes.

At the cochlear level there are several G protein-coupled receptors, whose activation causes protection against the ototoxic effects of cisplatin. Adenosine A1 receptors (A1AR) are in the vascular stria, in the spiral ganglion cells and in the organ of Corti. The largest percentage of these receptors are in the inner hair cells and Deiter’s cells, with lower levels in the outer hair cells. The otoprotective effects of A1AR consist in reducing oxidative stress by activating AOE antioxidant enzymes and suppressing NOX3 induction. The localized administration of an A1AR agonist would lead to enhanced activity of the antioxidant enzymes glutathione peroxidase and superoxide dismutase [26,27]. This would reduce hair cell damage and therefore the degree of hearing loss. In addition to these, recent data emphasize the anti-inflammatory role of A1AR at the cochlear level by suppressing the NOX3 isoform of NADPH and the STAT1-mediated inflammatory pathway (pathway with an essential role in ototoxicity) [28]. It should be noted that once generated, ROS will activate the STAT1 signal transducer that stimulates inflammation. If STAT1 and p53 (p53 protein) are activated, they together promote the process of cellular apoptosis. EGCG (Epigallocatechin-3-gallate) is a STAT1 inhibitor with an antioxidant effect that protects against cisplatin-induced hearing loss [20,29]. (Figure 3) shows the mechanisms described above.

Cannabinoid receptors 2 (CB2) are found in the vascular stria, inner hair cells, and spiral ganglion cells of the cochlea. There are studies that support the otoprotective role generated by the activation of these receptors, since once activated they cause the inhibition of the STAT1 pathway and thus promote the inflammatory process [30]. The activation of CB2 receptors leads to an increase in the level of antioxidant enzymes (glutathione, superoxide dismutase and catalase) that neutralize free radicals, thus reducing the production of ROS. CB2 receptors are expressed on immune cells involved in the inflammatory response. The activation of these receptors inhibits the production of pro-inflammatory cytokines (IL-6, TNF-α) which play a role in cellular apoptosis [31]. CB2 receptors are also involved in modulating the local immune response. Their activation leads to the cessation of local production of immune cells (which prevents damage to cochlear cells) and slows down the recruitment of new immune cells. We can say that CB2 receptors have an anti-inflammatory, antioxidant, anti-apoptotic and immunomodulatory role, and the use of CB2 agonists could be a promising strategy for preventing chemotherapy-induced hearing damage [8,32].

### 2.3. Mechanisms of Cisplatin-Induced Ototoxicity at the Mitochondrial Level

#### 2.3.1. Mitochondrial Genomic Damage and the Associated Generation of ROS

Cisplatin exerts toxic effects particularly on cells with high energy demands. A key mechanism involved in this toxicity is mitochondrial dysfunction, as mitochondria are essential organelles responsible for energy production, calcium homeostasis, and the regulation of programmed cell death. Increasing evidence suggests that cisplatin directly impairs both the structure and function of mitochondria, thereby contributing to the development of ototoxicity [8,33].

One of the earliest observed effects is mitochondrial DNA (mtDNA) damage. Cisplatin penetrates the mitochondria and binds to their genetic material. Unlike nuclear DNA, which is protected by histones and supported by robust repair mechanisms, mitochondrial DNA is significantly more vulnerable [34]. Cisplatin forms cross-links between nitrogenous bases, disrupting the transcription of genes essential for the synthesis of proteins involved in the mitochondrial respiratory chain. As a result, normal oxidative phosphorylation is impaired, leading to direct consequences on cellular energy production [35].

Because of respiratory chain dysfunction, the production of adenosine triphosphate (ATP) declines. Cells with high energy demands, such as cochlear hair cells, are among the first to be affected. In advanced stages of mitochondrial impairment, cisplatin promotes the opening of mitochondrial permeability transition pores, leading to the release of cytochrome C and other pro-apoptotic factors into the cytoplasm [35,36]. These factors activate caspases and initiate the mitochondrial apoptotic pathway, contributing to programmed cell death. This mechanism is relevant not only in auditory toxicity, but also in cisplatin-associated renal and neurological injury [36].

In addition to genomic and biochemical damage, cisplatin also disrupts mitochondrial dynamics, the processes that regulate the shape, distribution, and functional integrity of these organelles. Exposure to this cytotoxic agent is associated with a pronounced tendency toward mitochondrial fragmentation, a phenomenon linked to severe cellular stress and an increased susceptibility to apoptosis. This structural imbalance contributes to metabolic dysfunction and the accelerated degeneration of vulnerable cells [34,36].

One of the most significant consequences of mitochondrial dysfunction induced by cisplatin treatment is the excessive generation of ROS. Under normal physiological conditions, ROS are produced as byproducts of mitochondrial respiration during the electron transport chain activity. Their levels are tightly regulated by endogenous antioxidant systems. However, cisplatin profoundly disrupts this redox balance, leading to oxidative stress and further cellular damage [34,35].

Cisplatin exerts its mitochondrial toxicity primarily through binding to mtDNA, thereby inhibiting the expression of proteins encoded by mtDNA that are critical for the proper operation of electron transport chain complexes. Dysfunction of these complexes leads to impaired electron flow, resulting in electron leakage and the premature reduction of molecular oxygen. This interaction promotes the excessive production of O_2_^−^, a prevalent form of ROS [25,34,35]. The accumulation of ROS further damages mitochondrial components and other cellular structures, leading to lipid peroxidation, protein oxidation, and additional mtDNA degradation. As a result, the cell experiences severe oxidative stress that surpasses its intrinsic antioxidant capacity, ultimately culminating in cell death via apoptosis or necrosis [8,36].

In conclusion, cisplatin profoundly affects mitochondria both through direct damage to the mitochondrial genome and by initiating a cascade of metabolic and oxidative reactions that compromise the cell’s energy-generating capacity. The involvement of mitochondria in cisplatin-induced toxicity highlights these organelles as promising therapeutic targets for tissue-protective strategies.

#### 2.3.2. Bcl-2 Family

The Bcl-2 protein family forms the mitochondrial apoptotic pathway, being involved in promoting cell survival or death. Among the members of this family, Bcl-2 and Bcl-XL promote cell survival, and Bax, Bak, Bcl-XS, Bid, Bad, and Bim cause apoptosis [7,37]. Maintaining the equilibrium between pro-apoptotic and anti-apoptotic proteins is crucial for proper cellular function. Cellular injury triggered by various harmful stimuli can disrupt the balance, favoring the activation of apoptosis. This process begins when pro-apoptotic proteins like Bax and Bid translocate from the cytoplasm to the mitochondria. Their movement initiates a cascade of events that compromise the integrity of the outer mitochondrial membrane, leading to a loss of mitochondrial membrane potential, increased ROS production, and the release of cytochrome c from the mitochondria into the cytoplasm [8,33]. The release of cytochrome C activates caspases (especially caspase-9 and caspase-3), enzymes that are responsible for the execution of apoptosis [21,25].

The Bcl-2 family is directly involved in the regulation of mitochondrial function. Anti-apoptotic proteins such as Bcl-2 play a crucial role in protecting mitochondria from oxidative stress by maintaining the integrity of the mitochondrial membrane. However, their expression is reduced in the presence of cisplatin. Conversely, pro-apoptotic proteins contribute to the formation of pores in the outer mitochondrial membrane, facilitating the release of pro-apoptotic factors that initiate the apoptotic process [37]. An increased expression of anti-apoptotic proteins (such as Bcl-2) can inhibit the activation of caspases, thus preventing apoptosis. In the case of cisplatin, these anti-apoptotic proteins are reduced, which allows caspases to be activated. Caspases activate a series of processes that lead to DNA fragmentation, degradation of cytoskeletal components and cell death. Cisplatin-induced hearing loss has been associated with high levels of Bax and low levels of Bcl-2 in the cells of the Organ of Corti, in the spiral ganglion and the lateral wall [8,37].

#### 2.3.3. Caspases

Stress signals in the mitochondria cause procaspase-9 to cleave to its activated form, namely caspase-9. Once activated, caspase-9 intervenes in the cleavage and activation of a downstream effector caspase, namely caspase-3, this process leading to the apoptotic destruction of cells. Cisplatin causes the destruction of cells at the cochlear level by activating the initiating caspase-9 and the effector caspase-3 [33,38]. Studies conducted on laboratory animals have shown that intracochlear infusion of inhibitory substances for caspase-9 and caspase-3 decreased the degree of destruction of cochlear cells and implicitly the degree of hearing loss [39]. Unlike the caspases already mentioned, there is another caspase, namely caspase-1, which is involved in initiating inflammatory immune responses through the formation of inflammasomes [36]. There is research that claims that the activation of caspase-1 induces hearing loss after cytomegalovirus (CMV) infection by stimulating inflammatory factors such as interleukin-1β (IL-1β) and interleukin-18 (IL-18) [40].

Thus, any substance that inhibits caspase-1 could be beneficial and prevent ototoxicity by inhibiting inflammation initiated by cisplatin at the cellular level. The activation of caspases 3 and 9, along with the cellular apoptosis determined by cisplatin, were also inhibited with the help of CB2 receptors that are found in cochlear cells and react to the action of cisplatin [31]. The process of apoptosis therefore involves the activation of caspases as a common, final pathway, which in turn is activated by other signaling pathways expressed upstream. Inhibiting one or more pathways could save hearing cells from apoptosis and restore hearing [8].

#### 2.3.4. P53

Once penetrated tumor cells, cisplatin forms cross-links with the purine bases of DNA, causing damage to it. This damage affects the mechanisms of DNA replication and repair, the end being very predictable: cellular apoptosis [41]. The apoptotic signal is triggered by the phosphorylation of p53, a tumor suppressor gene and key mediator of apoptosis induced by DNA damage. In response to cellular stress, a portion of activated p53 translocates to the mitochondria, where it interacts with pro- or anti-apoptotic members of the Bcl-2 family, either promoting or inhibiting their activity [42].

In addition to these effects, p53 contributes to the loss of mitochondrial membrane potential, promotes the release of cytochrome C, and activates caspase-3 at the mitochondrial level, thereby serving as a key initiator of apoptosis [39,42]. In cells with p53 deficiency it was observed that the release of cytochrome C and the activation of caspase-3 were not influenced so pronounced, therefore p53 acts upstream of mitochondrial apoptotic regulators [8]. Excess expression of Bcl-2 or Bcl-XL (with an antiapoptotic role) prevented the accumulation of p53 mediated by stressors, suggesting that there is a feedback link between p53 and mitochondrial apoptotic regulators [43].

The application of a p53 inhibitor, pifithrin-α, had beneficial effects on the auditory system by reducing p53 expression and decreasing caspase -1 and caspase -3 [44]. Systemic administration of the p53 inhibitor has otoprotective effects, but this mode of administration has negatively influenced the anti-carcinogenic effect of cisplatin, limiting its use in human treatment. This problem has been studied, and it has been observed by Benkafadar et al. that the local, intratympanic application of pifithrin-α protects auditory function, without having influenced the chemotherapy effect of cisplatin on tumor cells [43]. Further studies are needed to prove that pifithrin-α can be used safely in human agents without causing adverse effects.

### 2.4. Cisplatin Impact on Cellular DNA

The main targets of cisplatin’s ototoxicity are the outer hair cells, the vascular stria (the metabolic center of the cochlea), and the spiral ganglion cells. The mechanism of action consists of inhibiting cell division by blocking the replication of cellular DNA, a process due to the formation of ROS that damage DNA, ultimately leading to apoptosis [2,29]. Cisplatin undergoes an intracellular hydration process in cochlear cells. The cisplatin molecule, which is initially electrically neutral, loses 2 chloride molecules, thus becoming a highly reactive complex. This complex binds to the purine bases of DNA, especially guanine, resulting in a DNA-cisplatin complex [45]. The formation of this complex distorts the normal structure of DNA, causing the DNA strand to bend and twist, a process that prevents replication and transcription, causing double-stranded breaks that result in the cell cycle being stopped [14]. These structural changes cause DNA repair mechanisms, which try to eliminate the damage, but in the case of ototoxicity with cisplatin the repair processes are exhausted. The Nucleotide Excision Repair System (NER) attempts to detect and eliminate cisplatin adductions, but if the damage is too great, the system’s ability to correct these errors is compromised [7,14,21].

If DNA damage cannot be repaired, apoptosis signaling pathways are activated to remove damaged cells and prevent mutations from spreading. DNA damage activates several intracellular signaling pathways that cause apoptosis. The p53 protein is an important transcription factor that detects DNA damage. In the presence of cisplatin damage, p53 becomes active and triggers apoptosis [45]. DNA damage triggers the activation of pro-apoptotic proteins from the Bcl-2 family, such as Bax and Bak, which promote mitochondrial membrane permeabilization, resulting in the release of cytochrome C and the activation of caspases involved in the apoptotic process. Outer hair cells are particularly vulnerable to changes in DNA, their destruction leading to reduced hearing, and DNA damage in spiral ganglion cells causes dysfunctions in the transmission of the auditory signal to the brain, contributing to irreversible hearing loss [8,45].

Because cochlear cells do not proliferate, hearing loss is thought to be caused by DNA plating rather than destroying nuclear DNA [5]. Platinum DNA is concentrated in the nuclei of outer hair cells, support cells, marginal cells of the vascular stria, and cells in the spiral ligament. In cochlear cells there are enzymes that repair DNA, trying to compensate for its degradation process. DNA repair enzymes are called nucleotide excision repair enzymes (NERs) and are classified into 2 categories: transcriptionally coupled repair enzymes (TCRs) and global DNA repair enzymes (GDRs), each of which has a specific target [8]. Platinum-based compounds can be retained for a longer period in the vascular stria, leading over time to disorders of potassium homeostasis and endocochlear potential, both of which are important for optimal auditory function. Thus, changes in the cochlear metabolic balance occur, resulting in a delayed destruction of cochlear hair cells [5].

### 2.5. Role of Cytokines in the Ototoxicity Process

One of the mechanisms that contribute to the occurrence of ototoxicity in patients treated with cisplatin is oxidative stress and excess accumulation of ROS, as previously stated. If intracellular ROS levels could be decreased, the cells would be protected from cisplatin-induced apoptosis. Also, the high level of autophagy can lead to increased resistance of hair cells to the ototoxic effect supported by cisplatin. The process of autophagy and oxidative stress at the cellular level are extremely complex, being regulated by various pathways and circulating cytokines. How cytokines may influence the process of ototoxicity remains insufficiently researched. Understanding the role that cytokines play in cisplatin-driven ototoxicity can help identify new treatment targets [7,46].

Ying Xu and collaborators found an association between cisplatin-induced hearing loss and circulating cytokines. Thus, increased levels of cytokines M-CSF, IL-2RA and MIP-1β were determined in patients treated with cisplatin who experienced hearing loss. Cisplatin causes the destruction of inner ear cells, along with cochlear hair cells by generating and accumulating ROS. Several cytokines are involved in this process of cell destruction. The study initiated by Ying Xu analyzed 41 cytokines and observed a bidirectional relationship between cytokines and ototoxicity determined by cisplatin. IL-17 has been found to be involved in reducing the risk of hearing loss in children treated with cisplatin [46].

The mechanism by which IL-17 is supposed to intervene in the reduction in cisplatin-induced ototoxicity is represented by the amplification of autophagy. Studies show that the presence of autophagosomes and increased expression of genes involved in autophagy are associated with less damage to cochlear hair cells. Inhibitors of the autophagy process (such as chloroquine) can lead to amplification of hearing loss, causing severe damage [46]. PRDX1 (peroxiredoxin-1) acts as an autophagy activator through the PTEN-AKT signaling pathway, helping to prevent cisplatin-induced damage in spiral ganglion neurons [47]. Additionally, IL-17 has been found to stimulate the expression of key proteins involved in the autophagy process, such as Beclin-1 and LC3. It has also been shown that in hepatocellular carcinoma autophagy caused by IL-17 confers resistance to oxaliplatin. This provides a guarantee that an identical mechanism exists in cochlear hair cells that are exposed to the harmful effects of cisplatin [48]. IL-17 could be the saving solution by increasing the level of autophagy in cochlear hair cells, thus reducing the degree of ototoxicity. This is only a hypothesis, and more advanced research and thorough investigations are needed to confirm it.

Macrophage colony-stimulating factor (M-CSF), also referred to as colony-stimulating factor 1 (CSF1), is a cytokine released by osteoblasts. It serves as a hematopoietic growth factor that regulates the proliferation, differentiation, and survival of monocytes, macrophages, and progenitor cells in the bone marrow. M-CSF also plays a role in platelet recovery after chemotherapy treatment with cisplatin/carboplatin in patients with ovarian cancer [49]. In this sense, studies support the hypothesis that increased levels of M-CSF may be associated with the body’s resistance to cisplatin. Studies have shown that M-CSF may play a protective role against cisplatin-induced ototoxicity. M-CSF plays a role in the regeneration and survival of auditory cells and nerve cells in the inner ear, aiding in their protection against damage caused by cisplatin [49]. Specifically, M-CSF may be involved in reducing inflammation and oxidative stress produced by cisplatin in hearing cells, as well as promoting their regeneration after exposure to cisplatin [46]. Thus, exogenous administration or stimulation of endogenous M-CSF production could be a potential strategy for preventing or reducing cisplatin-induced ototoxicity during cancer treatment. However, it is important to carry out more research to fully understand the mechanisms involved and to develop effective and safe therapeutic strategies in this regard.

## 3. Otoprotective Strategies

It can easily be seen that the structures of the inner ear are the most susceptible to the deterioration produced by cisplatin. It should be noted that the most important damage is observed at the level of the outer hair cells in the basal part of the cochlea. This impairment initially leads to increased hearing thresholds for high frequencies, followed by progressive loss of low frequencies as treatment continues and affects other structures [50,51].

There are many promising drugs, but their effectiveness on the human species is still being studied. The main problem in this regard would be the route of administration because the systemic administration of these drugs could compromise the chemotherapy treatment on the tumor. The safest way in which the antitumor efficacy would not be affected is represented by the local administration of the medication, at the cochlear level, through a minor surgical procedure [8]. Thus, a good part of the medication with otoprotective potential that targets the mechanisms presented above could be used safely. Table 1. shows the potential drug targets proposed over time for the treatment of cisplatin-induced ototoxicity and is adapted from Sheth et al., 2017 [8].

Cochlea is endowed with cellular mechanisms that contribute to otoprotection. These mechanisms encompass endogenous antioxidant enzymes and compounds, heat shock proteins, renal cell adhesion molecule 1, anti-apoptotic proteins, as well as hormone and G protein-coupled receptors. However, if these mechanisms are present, why does the phenomenon of ototoxicity occur? It is believed that during cisplatin treatment, especially at high doses, these protective mechanisms become overwhelmed [21,51] and are no longer able to counteract the drug’s toxicity effectively [21].

Numerous studies have explored treatments with potential to prevent or reduce cisplatin-induced ototoxicity, targeting key molecules and pathways involved in the process. Current approaches are promising and open new research directions for preventing hearing loss [7]. Preclinical studies have demonstrated beneficial effects and otoprotective roles for certain therapeutic targets, but progression to clinical trials has been limited by insufficient funding, small patient cohorts, and lack of technical support, preventing firm conclusions about their applicability in patients [50]. Antioxidants are of particular interest in ongoing research, and it is hoped that future developments will lead to effective otoprotective drugs [21,51]. Table 2 summarizes the potential therapeutic agents, and the main drugs proposed in the treatment of cisplatin-induced ototoxicity, discussed in various stages of research, most of which have been proven only in preclinical studies.

Otoprotective agents usually contain sulfur or sulfhydryl groups, known as antioxidants and strong heavy metal chelators. There are numerous otoprotective agents mentioned in the literature that have been used in clinical trials: amifostine, L-methionine, lipoic acid, methyl thiobenzoic acid, sodium thiosulfate, glutathione ester, melatonin, vitamin E, N-acetylcysteine (NAC), resveratrol [8]. A study conducted on rats found that N-acetylcysteine provided protection against ototoxicity induced by cisplatin [62,63]. STS has also proven to be effective in reducing cisplatin-induced ototoxicity [66,68]. However, there are some reservations in their use. For example, for STS, it was observed that with its systemic administration, the cisplatin-STS complex is formed, which reduced the circulating level of cisplatin, leading to an ineffective antitumor therapy [72]. So, these substances should be administered locally for effective otoprotection.

Another sulfur-containing compound with an otoprotective role is D-methionine. In animal studies, this compound has shown its otoprotective effects at the cellular level by enhancing the activity of antioxidant enzymes, both through systemic administration and local delivery at the round window [69,71,72]. Dexamethasone has demonstrated an otoprotective role in animal studies after transtympanic administration, but in clinical studies the results are questionable, the otoprotective effect being limited in intratympanic administration [7]. High dose of amifostine provided otoprotection in a study on hamsters, but a neurotoxic effect was also observed in its use [73]. Substances with promising results for otoprotection include lipoic acid, ebselen, diethyldithiocarbamate and 4-methylthiobenzoic acid [8].

Unlike the preclinical studies that gave results, mentioned earlier, the studies in humans looked a little different. Regarding amifostine, there are studies that do not find it effective as an otoprotector in patients with metastatic melanoma, neuroblastoma or germ cell tumors treated with cisplatin, [106] but there are also studies that support that high doses of this drug can provide a significant otoprotective role [74,75]. N-acetylcysteine is involved in the capture of ROS, but also in the synthesis of the antioxidant glutathione. Human studies have shown ambiguous results regarding transtympanic administration, and further studies are needed to observe its effects [64,65].

The FDA has recently approved STS as the first treatment aimed at preventing cisplatin-induced hearing loss in children and adolescents diagnosed with solid tumors that are localized and non-metastatic [6]. The STS approval was made based on two phase 3 clinical trials (SIOPEL 6 and COG ACCL0431) [66,67]. These studies showed a significant decrease in the incidence of hearing loss among children aged between 1 month and 18 years. STS is involved in the capture of ROS and causes an increase in the level of endogenous antioxidants, favoring their production. Another mechanism of action is the direct interaction with cisplatin molecules, being able to neutralize its ototoxic effects. The administration of STS after treatment with cisplatin allows it to act on tumor cells, without disrupting its anti-cancer function [1,6,7].

The SIOPEL 6 study was conducted on 109 pediatric subjects with hepatoblastoma who received 20 g/m 2 intravenous STS 6 h after discontinuation of cisplatin treatment during 4 preoperative courses and 2 postoperative courses. It was observed that STS significantly reduced hearing loss in these patients by 48% (only 18 children out of the 55 who received STS had hearing loss, compared to the control group that did not receive STS where 29 out of 46 children had hearing loss). This study also signaled the potential adverse effects recorded during administration, so there were 16 side effects of which 8 were attributed to STS. Among these adverse effects were mentioned: infections, neutropenia, anemia, nausea, tumor progression [66].

The otoprotective effect of STS has been associated with a high sodium load which must be considered when planning therapy. To avoid adverse effects, this agent should be administered with the utmost caution and the patient should be monitored during administration. There are some limitations, however, because there is not yet a large, multi-institutional study in large batches of patients or in batches of adults to demonstrate its effectiveness. The potential of STS has only been demonstrated in a subgroup of pediatric patients with a specific type of cancer, which limits its use [107,108].

Apart from STS, none of the mentioned agents are used in clinical practice, as current studies have not proven their definite benefit in preventing ototoxicity. Both gene therapy for susceptible patients, as well as the local transtympanic administration of protective drug agents acting on the inner ear, are areas of interest for current and future research, to maintain the effectiveness of chemotherapy to obtain an effective otoprotective effect [1].

Cisplatin remains a life-saving treatment for many types of cancer, and its discontinuation is not a viable option. However, by understanding the precise mechanisms underlying its adverse effects, it becomes possible to reduce toxicity without compromising its antitumor efficacy. The identification of therapeutic targets allows for the development of interventions aimed at counteracting its harmful effects. These targets enable early and personalized strategies to protect auditory structures [50].

Table 1 highlights the potential therapeutic targets proposed over time. These include various receptors, transporters, and pro-inflammatory markers on one side, and antioxidant molecules on the other. Inhibiting the molecules with deleterious roles, or enhancing those with antioxidant properties, may be the key to mitigating cisplatin-induced ototoxicity. Both older and more recent studies have investigated the mechanisms of action of these molecules in the development of potential protective agents. We believe that a clear understanding of these key molecules and their pathways significantly increases the likelihood of discovering effective drugs that prevent cisplatin-related hearing loss. Priority in both clinical and replication studies should be given to molecules involved in antioxidant defense systems. Numerous studies have supported their efficacy, with references provided for each molecule in Table 1. Although the interest in this field is evident, large-scale human studies are still required to determine their real-world effectiveness [7,50,51].

Table 2 complements Table 1 by listing substances and drugs currently under investigation at various stages of development. Most of these agents exhibit anti-inflammatory and antioxidant properties and target the molecular pathways described in Table 1. Table 2 also includes substances that inhibit specific cellular pathways, as well as compounds with complex mechanisms of action. The studies supporting their efficacy are either in preliminary phases, not yet conducted on human subjects, or limited by small sample sizes and lack of replication. Therefore, it remains uncertain whether these agents will ultimately be suitable for clinical use in patients with cisplatin-induced ototoxicity.

One of the most studied pathogenic mechanisms in cisplatin-induced ototoxicity is oxidative stress. This redox imbalance triggers a vicious cycle of cellular destruction, making the endogenous antioxidant system a major therapeutic target. In addition to oxidative stress, another important mechanism involved in ototoxicity is the activation of mitochondrial apoptotic pathways. Relevant therapeutic targets in this direction include molecules from the Bcl-2/Bax family, which are involved in regulating mitochondrial permeability. Substances such as resveratrol and curcumin have been investigated for their ability to block these apoptotic pathways, thereby reducing cellular damage in the inner ear [36,37].

Inflammation represents a third pillar in the pathogenesis of ototoxicity [54]. Cisplatin activates inflammatory factors such as NF-κB and increases levels of pro-inflammatory cytokines (TNF-α, IL-6), contributing to cochlear structure destruction [55]. In this context, corticosteroids like dexamethasone have been shown to be effective in reducing inflammation. Additionally, biological therapies such as etanercept (TNF-α inhibitor) may represent viable therapeutic options for combating local inflammation.

Specific transporters such as CTR1 and OCT2 facilitate the intracellular uptake of cisplatin. Inhibition of these transporters may reduce cisplatin accumulation in the inner ear without compromising its systemic antineoplastic efficacy [11,12]. In this context, drugs like cimetidine (an OCT2 inhibitor) and metformin (which modulates transporter activity) offer a novel perspective in auditory protection.

Currently, most of these treatments remain in the research phase or are used off-label, highlighting the urgent need for approved therapies and robust clinical trials. A deeper understanding of the molecular mechanisms involved in cisplatin-induced ototoxicity has enabled the identification of clear therapeutic targets, paving the way for the development of effective and personalized treatments. Interventions targeting oxidative stress, apoptosis, inflammation, cellular transport, and tissue regeneration have the potential to significantly improve the management of this condition, contributing to the preservation of quality of life in oncology patients.

## 4. Cisplatin-Induced Ototoxicity Monitoring

**Figure 4:**
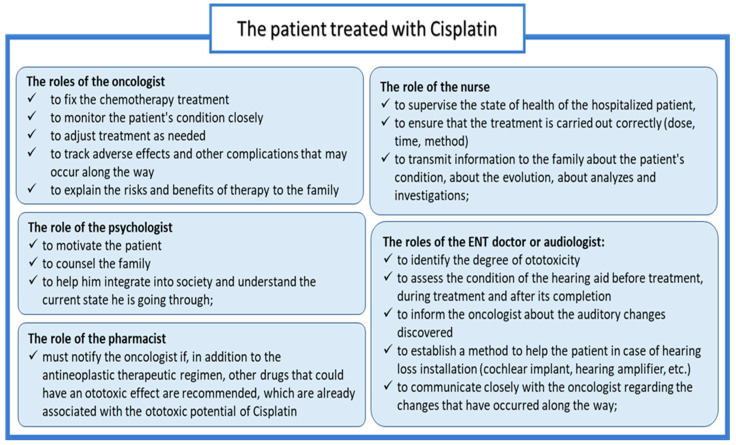
The role of the multidisciplinary team in the management of patients exposed to Cisplatin [1,109].

Prospective and early audiological evaluation remains the most reliable and handy method for identifying ototoxicity before it becomes symptomatic. This is where the role of the multidisciplinary team to manage the case comes in, so that the results are for the benefit of the patient. (Figure 4) illustrates the members of the multidisciplinary team, as well as the roles assigned to them.

There are countries that do not have management guidelines for patients at risk of ototoxicity who receive chemotherapy medication. In this case, the “Guide for the Audiological Management of People Receiving Cochleotoxic Drug Therapy”, developed by ASHA, can be extremely useful in implementing an ototoxicity monitoring program [1,109]. It is not easy to implement such a program, and for it to be accepted and used, it must contain clear, effective and cost-effective ototoxicity identification techniques, consider the National Health Care System, as well as the population to which it is addressed. The ototoxicity monitoring program must also consider the patient’s ability to respond to treatment, the patient’s level of alertness for good collaboration, and the most appropriate times during the protocol when hearing tests can be performed. Hearing tests should contain basic assessments, followed by monitoring and post-treatment assessments. The time interval between these tests may be different depending on the type of cancer, the dose given of cisplatin, the frequency of repetition of the dose and the combination with other drugs [1,109].

The audiological evaluation should include a detailed case history, followed by otoscopic examination, tympanometry, behavioral audiometry, vocal audiometry, pure-tone audiometry, testing of otoacoustic emissions and distortion product otoacoustic emissions (DPOAE), as well as high-frequency audiometry (HFA). These procedures are recommended to be performed for the baseline assessment, prior to the start of treatment, and at the 6-month follow-up assessment [1]. Audiological monitoring during treatment and evaluation at 1 month and 3 months includes otoscopy, behavioral audiometry, and conventional pure tone audiometry [110]. Only objective tests such as otoscopy, tympanometry, acoustic reflexes, and DPOAE or ABR (Auditory Brainstem Response) are effective and appropriate in evaluating patients who are either too small to respond or too weak due to disease. The most important tests for the early detection of ototoxicity are considered HFA and DPOAE, but like any test they also have some limitations [1,110,111]. Table 3. describes the advantages and disadvantages of these procedures.

Cisplatin-induced ototoxicity monitoring should be comprehensive, regular and tailored to the needs of each patient. It involves objective tests (audiometry, OAE), evaluation of risk factors, monitoring of subjective symptoms and adjustment of treatment according to the results obtained. The main purpose of monitoring is to detect any changes in hearing function early and intervene in a timely manner to prevent irreversible hearing loss [1].

If we were to outline the essential aspects aimed at the optimal monitoring of ototoxicity caused by cisplatin, we should first point the evaluation of auditory function before starting treatment. This evaluation is extremely important because it helps us determine the point from which we start. There are patients with pre-existing hearing loss who may be influenced later by treatment with cisplatin. The pre-treatment assessment should include baseline audiometry to evaluate hearing thresholds across various frequencies—particularly high frequencies, which are more vulnerable to cisplatin-induced damage. It should also involve tympanometry to exclude other potential causes of hearing loss, as well as otoacoustic emissions (OAE) testing, a non-invasive and sensitive method for identifying early auditory changes [110,117].

During the treatment, the investigations should be repeated at an interval established by the oncologist and the otolaryngologist to observe especially the high frequencies, which are the first to be affected by ototoxicity. Ideally, the patient should be tested before each dose of cisplatin received. Also, the patient should be asked if he reports auditory symptoms such as: difficulty understanding language in noisy environments, decreased hearing acuity, vertigo, tinnitus, feeling of fullness in the ear. After completion of cisplatin treatment, monitoring should continue to detect any late effects [115].

Hearing evaluations should be conducted at regular intervals following the completion of chemotherapy, as hearing loss may be progressive and can emerge even months after treatment has ended. Monitoring long-term auditory function is especially important in children, who are more vulnerable to ototoxicity, as hearing impairments can significantly impact speech and language development. Post-treatment assessments should be scheduled at 1, 3, 6, and 12 months after chemotherapy, and then annually as necessary [1,109,114].

## 5. Limitations, Conclusions, and Future Perspectives

Although the mechanisms that lead to ototoxicity are known, they are extremely complex, comprising numerous structures and molecules. Some of them could represent extremely important therapeutic targets that block the ototoxicity cascade and prevent hearing loss. Despite the many promising drugs targeting these mechanisms, their efficacy on the human species remains under study, and the main problem is a route of administration that does not affect antineoplastic therapy. It is proposed that otoprotective agents be administered locally to avoid compromising the anti-cancer treatment by drug interaction in the case of systemic administration. Although there are many antioxidants and otoprotective agents with promising results, their effectiveness is not yet proven in clinical practice, and human studies have shown conflicting results.

Ototoxicity is a side effect that permanently affects the quality of life of cancer patients. All the molecular or cellular mechanisms presented, involved in the ototoxicity process determined by the treatment with cisplatin, highlight the complexity of this process, but also the difficulty of finding effective otoprotective agents that can be safely used in humans. The patient approach in the multidisciplinary team, including audiological monitoring of the dynamics of the auditory status, is essential for the prevention or limitation of ototoxic effects and for the early diagnosis and treatment of these effects, with direct benefits on the quality of life of patients.

The translation of preclinical findings into clinical practice remains a major challenge in the field of cisplatin-associated otoprotection. The molecular mechanisms involved in ototoxicity (such as oxidative stress, inflammation, intracellular Ca^2+^ accumulation, caspase activation, and disruption of mitochondrial transport) have been extensively documented in animal models [118]. However, applying this knowledge to human medicine requires further validation. Although compounds like D-methionine, ebselen, and TRPV1 channel inhibitors have demonstrated efficacy in preventing hearing loss in animals, the lack of reliable predictive models for pharmacokinetics and pharmacodynamics in humans represents a significant obstacle [119].

Pharmacological and immunological differences between species used in preclinical studies and human patients reduce the direct relevance of many experimental findings. For example, in murine models, administration of D-methionine led to over a 75% reduction in hair cell loss (*p* < 0.001), yet these results have not been similarly replicated in human studies [118]. Moreover, interindividual variations in drug metabolism and enzymatic activity (such as polymorphisms in the GSTP1 gene involved in cisplatin detoxification) can profoundly influence therapeutic response and the risk of ototoxicity [120].

Implementation strategies in clinical practice must consider the oncological context of the patient and the critical need not to compromise cisplatin’s efficacy. A partial success story is exemplified by the multicenter ACCL0431 study (Children’s Oncology Group), in which systemic administration of STS reduced the incidence of ototoxicity in children from 56.4% to 28.6% (*p* = 0.004), without affecting overall survival in non-metastatic tumors [67]. However, in the subgroup with metastatic tumors, 3-year survival was lower in the treated group (84% vs. 97%, *p* = 0.04), highlighting the potential risks of non-specific systemic protection.

The choice of administration route (local versus systemic) is a crucial strategic element in clinical translation. Transtympanic administration of dexamethasone or N-acetylcysteine type agents in pilot studies has resulted in elevated concentrations within the perilymph, with demonstrated otoprotective effects and without significant systemic interactions [120]. Nevertheless, this method presents logistical challenges, requires specialized equipment, and may be difficult to implement on a large scale, particularly in pediatric oncology.

One of the most significant challenges is the biological differences between experimental models and human organisms. Many of the beneficial effects observed in murine models, for example, do not always translate into positive outcomes in humans due to differences in drug metabolism, biodistribution, and immune response. Moreover, the doses of cisplatin and the timing of protective treatments in preclinical studies are often artificially optimized to maximize protection, which is not always feasible in the clinical setting, where therapeutic regimens are tailored to individual oncological needs [119,120].

Beyond efficacy, the long-term safety of otoprotective agents is essential. For instance, although amifostine has demonstrated effectiveness in preclinical studies, it has been associated with significant adverse effects in humans (including severe hypotension and nausea, with an incidence exceeding 40%) which has limited its applicability in clinical practice [119]. Additionally, the potential for protecting tumor cells against oxidative stress represents a valid concern and has sparked numerous ethical and scientific controversies.

The lack of comparative quantitative data represents a significant limitation in many clinical studies investigating strategies to prevent cisplatin-induced ototoxicity. A recent meta-analysis estimated a global prevalence of cisplatin-associated hearing loss at 49.21% (95% confidence interval: 42.62–55.82%). However, the authors emphasized that most of the included studies did not report *p*-values, or the number needed to treat (NNT) to prevent one case of hearing loss. This lack of rigorous quantitative data impairs the ability to objectively compare the effectiveness of different interventions and limits the development of standardized, evidence-based clinical protocols [121].

Integrating findings from in vitro and animal studies into human treatment protocols requires a series of carefully controlled clinical trials, ideally conducted in multiple phases and involving multidisciplinary teams. The inclusion of pharmacogenomic data may also improve patient stratification and contribute to a better understanding of interindividual variability in response to protective agents. Translational medicine must, therefore, be adaptive and iterative, constantly integrating clinical feedback to refine experimental hypotheses and therapeutic strategies.

One proposed solution involves the development of standardized clinical protocols that include baseline and periodic audiological assessments, patient stratification based on genetic risk factors (e.g., TPMT, GST polymorphisms), personalized selection of otoprotective agents, and the use of advanced formulations (e.g., nanoparticles, PEGylated carriers) [119,121]. The implementation of such protocols requires multidisciplinary collaboration and the integration of clinical research into routine practice.

It is also essential that any proposed therapeutic intervention does not compromise the cytotoxic efficacy of cisplatin against the tumor. This is a major concern when testing adjuvant agents: antioxidant drugs, for example, may reduce oxidative stress not only in healthy cells but also in tumor cells, thereby diminishing the effectiveness of cancer treatment. Therefore, any protective agent must be carefully tested to ensure it acts selectively in vulnerable tissues (such as the cochlea or kidneys) without interfering with cisplatin’s antitumor activity.

Another limiting factor is the lack of validated clinical biomarkers that could enable monitoring of the protective therapy’s effectiveness or the risk of ototoxicity in each patient. Without precise tools for evaluation and prediction, the personalized application of these treatments remains challenging.

Finally, clinical implementation requires an extended development period—from phase I trials assessing safety and pharmacokinetics, to phases II and III, which focus on efficacy and comparison with the standard of care. This process involves significant investments, considerable time, and often interdisciplinary collaboration between oncologists, audiologists, molecular researchers, and the pharmaceutical industry.

Systemic administration is generally easier to implement clinically, especially for patients already receiving intravenous chemotherapy. This route allows the protective agent to reach the entire body, including the inner ear, and can be relatively easily integrated into standard therapeutic regimens [51].

On the other hand, local administration—such as transtympanic injection into the middle ear or direct application into the perilymphatic fluid—offers a major advantage: the concentration of the active substance at the target tissue, with reduced systemic exposure and a lower risk of interfering with oncologic treatment. This approach can help avoid unwanted effects on tumor cells or other organs. Compounds such as D-methionine or TRPV1 channel inhibitors have been tested using these methods, yielding promising results in animal models. However, the practical applicability of these approaches is limited by several factors: the procedures can be invasive, require specialized equipment and appropriate training, and repeated administrations may be difficult, especially in pediatric or immunocompromised patients [56,69,70].

Hybrid strategies such as nanoparticle-based delivery systems or slow-release formulations may offer a future solution, balancing both efficacy and safety. Ultimately, the decision should be individualized and supported by robust clinical evidence.

The safety of these therapies also depends on their specific pharmacokinetics. In the case of systemic administration, it is essential that the drug does not reach toxic concentrations in other sensitive organs, such as the liver, kidneys, or central nervous system. In contrast, local administration can avoid these issues but raises other challenges, such as ensuring adequate diffusion within the cochlea and maintaining an effective concentration throughout the duration of therapy. Another important aspect is the timing of administration in relation to chemotherapy. For systemically administered agents, timing is crucial to avoid interfering with the cytotoxic activity of cisplatin [118,120].

One of the main factors contributing to the discrepancies between preclinical and clinical studies is the difference in dosage and administration. In preclinical studies, the doses of NAC or amifostine are often adjusted to achieve optimal protection, administered in a simpler and more controlled regimen, and timed precisely to maximize their effectiveness. In contrast, in clinical studies, the administration of these agents takes place in a much more complex context, where multiple factors must be considered: the patient’s general condition, tumor type, chemotherapy regimen, as well as the synchronization between chemotherapy and the protective treatment. These variables make it difficult to optimize dosing and timing, resulting in less predictable treatment efficacy.

Another significant factor influencing clinical outcomes is the individual variability among patients. Even within a population of patients with the same conditions, there are substantial differences in drug metabolism and response. For example, NAC, an antioxidant that counters oxidative stress, may have varying impacts depending on the patient’s overall health status, the presence of comorbidities (such as liver or kidney disease), and concurrent treatments. Additionally, NAC metabolism can differ from one patient to another, meaning that some individuals may experience otoprotective benefits, while others may not respond as effectively [63,64,65].

In the case of amifostine, which protects healthy cells through the activation of the enzyme thioredoxin reductase, clinical results have been contradictory partly due to its associated toxicity profile. While amifostine has shown significant protective effects in preclinical studies [73], clinical trials have reported a higher incidence of adverse effects such as nausea, vomiting, and hypotension [74,75]. Although these side effects are not severe in all cases, they can significantly reduce patient adherence to treatment and interfere with the chemotherapy regimen, leading to ambiguous outcomes. Furthermore, amifostine administration requires careful monitoring of the patient’s condition, and its timing in relation to cisplatin is essential to maximize protection without compromising antitumor efficacy.

These inconsistencies underscore the need for a more personalized approach in ototoxicity prevention, where protective agents are selected and dosed based on each patient’s genetic, metabolic, and oncologic profile. Further research is essential to develop predictive biomarkers and stratification algorithms that can guide therapeutic decisions and improve clinical outcomes.

Another reason why clinical studies on these agents have yielded ambiguous results is the difference in methodological approaches. While in preclinical studies it is relatively easy to control the timing and dosage of protective agent administration, this is much more difficult to achieve in clinical trials. Additionally, clinical studies often include large numbers of patients with various types of cancers and diverse demographic characteristics, resulting in significant heterogeneity in treatment response. Genetic factors, such as polymorphisms in genes involved in cisplatin metabolism and oxidative stress response, can significantly influence the effectiveness of protective agents, further complicating the interpretation of results [2,3].

Cisplatin-induced ototoxicity represents a major clinical challenge, arising from a complex interplay of factors that include individual susceptibility, the pharmacokinetic profile of the drug, and the treatment regimen. Among patient-related risk factors, age is a critical determinant: children, particularly those in the language development period, are highly vulnerable since hearing loss has profound cognitive and educational consequences [5,122]. Renal function is another key determinant, as cisplatin is eliminated primarily via the kidneys; reduced creatinine clearance is associated with drug accumulation and prolonged tissue exposure [123]. Additionally, genetic polymorphisms in genes involved in cisplatin transport (e.g., SLC22A2/OCT2) or antioxidant defense (SOD2, GSTP1) have been linked to higher ototoxicity risk, explaining the interindividual variability observed in clinical practice [108,122].

Another central aspect is the relationship between dose and toxicity. Several clinical studies have demonstrated a cumulative dose-dependent effect, with a significant increase in ototoxic risk once exposure exceeds 200–300 mg/m^2^, and a very high incidence of severe hearing loss beyond 400 mg/m^2^. However, this relationship is not strictly linear, as it is influenced by the frequency and mode of administration (large, intermittent doses versus fractionated dosing) as well as by concomitant exposure to other ototoxic agents, such as aminoglycosides or calcineurin inhibitors. Thus, cumulative dose serves as an orientative marker but is insufficient to accurately predict individual risk [111,122].

Recent findings suggest that the true mechanism of ototoxicity extends beyond a simple dose–effect relationship. Cisplatin has a unique ability to bind to proteins and intracellular structures within the cochlea, leading to persistent platinum deposits that can remain for months or even years after treatment completion. This prolonged retention explains why hearing loss may progress even after chemotherapy has ended, emphasizing that not only plasma levels but also local distribution and persistence are critical factors in understanding toxicity [1,8,122].

In this context, the concept of therapeutic drug monitoring (TDM), so valuable in the case of aminoglycosides or calcineurin inhibitors, becomes considerably more difficult to apply for cisplatin. While aminoglycosides show a clear correlation between plasma concentrations and nephro- or ototoxic risk, cisplatin exhibits a much weaker association, since its active and toxic forms are not fully represented by the free plasma fraction. The local concentration in the perilymph and hair cells appears to be the true determinant of ototoxicity, yet this parameter cannot be directly monitored in clinical practice. For this reason, classical TDM has not become routine for cisplatin, although experimental studies suggest that individualized pharmacokinetic monitoring could provide benefits for high-risk patients.

Nevertheless, TDM principles can be indirectly applied through integrated risk monitoring. Adjusting doses according to creatinine clearance, avoiding excessive cumulative exposure, and introducing validated protective measures (such as delayed sodium thiosulfate administration in pediatric oncology) are practical strategies. For example, in the SIOPEL-6 trial, administration of sodium thiosulfate six hours after cisplatin significantly reduced the incidence of hearing loss from 63% to 33% (*p* < 0.001), without compromising overall survival, thus confirming the feasibility of a pharmacokinetically timed protective strategy [66,67]. In parallel, periodic audiometric testing and the use of oxidative stress biomarkers can serve as “clinical proxies” for a TDM system adapted to the unique characteristics of cisplatin. Cisplatin ototoxicity depends not only on cumulative dose but also on individual patient characteristics and intracochlear drug accumulation. While classical TDM is not readily applicable, a modern, multidimensional approach—integrating adjusted pharmacokinetics, susceptibility biomarkers, and early functional monitoring—offers a realistic path toward reducing risk without compromising oncologic efficacy.

We aim to encourage clinical studies that aspire to identify effective therapeutic strategies to successfully counteract ototoxicity. Although our center does not currently possess the necessary resources to initiate such studies, we firmly believe that valuable results will eventually emerge from other research centers. We advocate for multidisciplinary collaboration and effective communication to ensure efficient and promising management of ototoxicity in oncology patients. There is an urgent need to implement standardized and internationally recognized protocols for the monitoring and evaluation of these patients, as many countries currently lack specific guidelines for ototoxicity surveillance in oncological care.

By gaining a thorough understanding of the mechanisms underlying ototoxicity, identifying therapeutic targets, and conducting clinical trials that demonstrate the efficacy of protective interventions, we stand a real chance of improving the quality of life for oncological patients and ensuring a return to normal life after overcoming the critical treatment phase.

## Figures and Tables

**Table 1:** Potential drug targets in the treatment of Cisplatin-induced ototoxicity.

Drug Targets		Mechanisms of Action	Bibliographical Sources
RECEPTORS	Adenosine A1 receptor (A1AR)	Drugs targeting this receptor could enhance endogenous antioxidant defenses while suppressing the inflammatory NOX3/STAT1 pathway	[26,27,28,52]
	Cannabinoid 2 receptor (CB2)	Anti-apoptotic effect	[30,31,32]
TRANSPORTERS	Organic cation transporter 2 (OCT2)	Involved in the mechanisms that regulate the cellular uptake of cisplatin	[12,13,53]
	Copper transporter 1 (CTR1)	Plays a role in facilitating the entry of cisplatin into cells	[10,11]
PRO-INFLAMMATORY MARKERS	Tumor necrosis factor-α (TNF-α)	A cytokine that promotes inflammation, triggered by cisplatin	[54]
	Signal transducer and activator of transcription-1 (STAT1)	A pro-inflammatory transcription factor	[20]
	Nuclear factor-kB (NF-kB)	A transcription factor that promotes inflammation and apoptosis	[38,55]
	Transient receptor potential vanilloid 1 (TRPV-1)	A marker of oxidative stress and inflammation at the cochlear level, facilitating the entry of cisplatin	[19,56]
ANTIOXIDANT DEFENSE SYSTEM	NOX3	Generates ROS in cochlear cells	[17]
	Superoxide dismutase (SOD)	Plays a role in converting the superoxide anion into hydrogen peroxide (H_2_O_2_) and O_2_	[57]
	Catalase (CAT)	Converting H_2_O_2_ into H_2_O and O_2_	[57]
	Glutathione (GSH)	Endogenous antioxidant molecule	[24]
	Glutathione peroxidase (GSH-Px)	Catalyzes the breakdown of H_2_O_2_ into H_2_O and O_2_ by using GSH	[57]
	Glutathione reductase (GR)	Plays a role in regenerating GSH from GSSG	[57]
	Glutathione S-transferase (GST)	Conjugate GSH with xenobiotics	[58]
	Kidney injury molecule-1 (KIM-1)	Oxidative stress marker in the cochlea	[59]
	Vitamin E	Antioxidant molecule	[60]
MISCELLANEOUS	Signal transducer and activator of transcription 3 (STAT3)	Plays a role in cytoprotection mechanisms	[61]

**Table 2:** Potential therapeutic agents and drugs in the treatment of Cisplatin-induced ototoxicity.

Therapeutic Agent		Mechanisms of Action	Bibliographical Sources
Antioxidant and anti-inflammatory treatments	N-acetyl cysteine (NAC)	Antioxidant molecule	[62,63,64,65]
	Sodium thiosulfate (STS)	Antioxidant molecule	[6,66,67,68]
	D -Methionine (D -Met)	Antioxidant molecule	[69,70,71,72]
	Amifostine	Involved in capturing free radicals	[73,74,75]
	Allopurinol	Xanthine oxidase inhibitor	[23]
	Ebselen	It is a glutathione peroxidase mimetic	[23,76]
	Dexamethasone	Anti-inflammatory effect	[77,78,79,80,81]
	Etanercept	Anti-inflammatory effect	[54,82]
	Statins	Antioxidant and anti-inflammatory effect	[83]
	Curcumin	Antioxidant, anti-inflammatory, anti-apoptotic effect	[84]
	Capsaicin	Antioxidant and anti-inflammatory effect	[32]
	Apelin-13	Antioxidant and anti-inflammatory effect	[85]
	Aucubin	Antioxidant and anti-inflammatory effect	[86]
	Astaxanthin	Antioxidant, anti-apoptotic effect	[87]
	R-phenylisopropyladenosine (R-PIA)	Anti-inflammatory effect	[28]
	Alpha-lipoic acid	Anti-apoptotic effect	[88]
	ROSI (ACSL4 inhibitor)	Inhibition of lipid peroxide production	[89]
	Inhalation of gaseous H2	Inhaling 2% H2 gas has antioxidant effects	[90]
	Avenanthramide-C (AVN-C)	Antioxidant and anti-inflammatory effect	[91]
Inhibitors of cellular pathway	Agmatine	Upregulates the PI3K/AKT pathway to attenuate apoptosis	[92]
	Rutin	Prevents apoptosis by activating the PI3K/AKT signaling pathway	[93]
	Eupatilin	Intervenes in the mitochondrial apoptosis pathway	[94]
	Puerarin	Suppresses ROS generation, modulates Bcl-2 family proteins, and influences the mitochondrial pathway of apoptosis	[95]
	Meclofenamic Acid	Inhibition of excessive autophagy induced by cisplatin.	[96]
	Trehalose	Provides protection against cisplatin-induced damage to cochlear hair cells through the activation of autophagy	[97]
	YTHDF1	Involved in the process of autophagy	[98]
Other novel mechanisms antagonizing Cisplatin ototoxicity	Combination of PFT-α, D-Met	Significant cell protective effect	[99]
	The conjugation of dexamethasone with nanoparticles	Enhances the distribution of the drug and boosts dexamethasone’s solubility and bioavailability	[100]
	Mannitol	Modulating the permeability of the blood-labyrinth barrier (BLB)	[101]
	Aspirin	Decreasing cochlear metabolic activity	[102]
	Cool	Reducing the absorption of cisplatin in cochlear cells, the production of reactive oxygen species, and inflammatory factors	[103]
	Salubrinal	Affects various cellular processes	[104]
	Acetophenone	Affects various cellular processes	[105]
	Pifithrin-α (PFT-α)	It is a p53 inhibitor	[43,44]
	Epigallocatechin gallate (EGCG)	STAT1 inhibitor	[61]

**Table 3:** Advantages and disadvantages of HFA and DPOAE.

	**Benefits**	**Disadvantage**
**HFA (>8 kHz)**	Is the most sensitive test for identifying ototoxic hearing loss [112,113]. Can identify hearing loss in the early stages, before symptoms become apparent in low frequencies [109]. Can be used in cases of drug toxicity or exposure to toxic substances to monitor treatment progress or evaluate preventive measures [114]. Some hearing losses, like those at high frequencies, may go undetected with conventional audiometry but can be identified using HFA [115].	The efficiency may be affected because HFA takes time [114]. Not standardized [115]. Not widely used due to the requirement for additional equipment, such as over-the-ear headphones [116]. Are more sensitive to ambient noise and external interference, which can affect testing accuracy [112,114]. Requires good patient cooperation to obtain accurate results. Young children or people with cognitive impairments may have difficulty completing the test [1].
	**Benefits**	**Disadvantage**
**DPOAE**	Is sensitive to subclinical cochlear dysfunctions, allowing the detection of hearing loss even in the early stages [115]. Is specific to cochlear activity and helps distinguish cochlear hearing loss from other causes [110]. Is completely non-invasive and painless, being suitable for patients of all ages, including newborns and young children [1]. Results are consistent and reproducible, allowing easy monitoring of hearing loss over time [109]. Can be used in neonatal hearing screening programs for early detection of hearing loss in newborns [117]. Can detect hearing loss earlier than standard pure-tone audiometry [117].	External interference, such as ambient noise or patient movement, can affect test results and lead to inaccurate interpretations [113]. May be less sensitive for low- or mid-frequency hearing loss or certain cochlear lesions [110]. There is no standard value for the criteria that indicate ototoxicity [1]. OAE has a limited frequency range (generally up to 8000 Hz) [110]. Accurate results require the patient to stay still, which can be difficult for young children or those with cognitive impairments [113]. Shows overall cochlear function but not the type or severity of hearing loss [115].

## Data Availability

No new data were created or analyzed in this study. Data sharing is not applicable to this article.

## Abbreviations

The following abbreviations are used in this manuscript:

A1AR	Adenosine A1 receptors
ABR	Auditory brainstem response
AOE	Antioxidant enzyme
CAT	Catalase
CB2	Cannabinoid receptors 2
CTR1	Copper transporter 1
CNS	Central nervous system
CMV	Cytomegalovirus
D-Met	D-Methionine
DPOAE	Otoacoustic emissions and distortion product otoacoustic emissions
EGCG	Epigallocatechin-3-gallate
FDA	Food and Drug Administration
GSH	Antioxidant reduced glutathione
GSH-Px	Glutathione peroxidase
GSSG	Oxidized glutathione
GR	Glutathione reductase
GST	Glutathione S-transferase
GDRs	Global DNA repair enzymes
HFA	High-frequency audiometry
H_2_O_2_	Hydrogen peroxide
IL-1 β	Interleukin-1β
IL-18	Interleukin-18
KIM-1	Kidney injury molecule-1
M-CSF	Macrophage colony-stimulating factor
NAC	N-acetyl cysteine
NOX3	Adenine dinucleotide phosphate oxidase 3
NADP^+^	Nicotinamide adenine dinucleotide phosphate
NER	Nucleotide excision repair system
NF-kB	Nuclear factor-kB
OCT	Organic cation transporter
OAE	Otoacoustic emission
P53	Tumor antigen p53
PFT- α	Pifithrin-α
PRDX1	Peroxiredoxin-1
ROS	Reactive oxygen species
SOD	Superoxide dismutase
STAT1	Signal transducer and activator of transcription 1
STAT3	Signal transducer and activator of transcription 3
STS	Sodium thiosulfate
SNP	Single-nucleotide polymorphism
TRPV1	Transient receptor potential vanilloid 1
TCRs	Transcriptionally coupled repair enzymes
TNF-α	Tumor necrosis factor-α
