# Correction: Iațentiuc et al. From Ototoxicity to Otoprotection: Mechanism and Protective Strategies in Cisplatin Therapy. *Pharmaceuticals* 2025, *18*, 1543

**DOI:** 10.3390/ph19020211

**Published:** 2026-01-26

**Authors:** Andreea Iațentiuc, Sebastian Romică Cozma, Otilia Elena Frăsinariu, Ingrith Crenguța Miron, Iustin Mihai Iațentiuc, Lucia Corina Dima-Cozma, Raluca Olariu, Anca Postolache, Ana-Maria Laura Buga, Alexandru Stingheriu, Edilene Boéchat, Oana Roxana Bitere-Popa

**Affiliations:** 1 Grigore T. Popa University of Medicine and Pharmacy, 700115 Iași, Romania; andreeacimpan@yahoo.com (A.I.); scozma2005@yahoo.com (S.R.C.); ingridmiron@gmail.com (I.C.M.); iatentiuc_iustin@yahoo.com (I.M.I.); cdimacozma@yahoo.com (L.C.D.-C.); raluca_bcn@yahoo.com (R.O.); ancap93@gmail.com (A.P.); anamaria_bnz@yahoo.com (A.-M.L.B.); alexandru.stingheriu@gmail.com (A.S.); oana.bitere@gmail.com (O.R.B.-P.); 2 Department of Audiology, Speech Pathology and Physiotherapy, Faculty of Human and Health Sciences, Pontifical University of São Paulo, São Paul 01246-904, Brazil; eboechat@pucsp.br

In the original publication [1], there was an error regarding the affiliations for Andreea Iațentiuc, Sebastian Romică Cozma, Iustin Mihai Iațentiuc, Lucia Corina Dima-Cozma, Raluca Olariu, Ana-Maria Laura Buga, Alexandru Stingheriu, Edilene Boéchat, and Oana Roxana Bitere-Popa.

We deleted the original affiliates 2, 3, 4, and 6, and we changed the original affiliate 1 to “1 Grigore T. Popa University of Medicine and Pharmacy, 700115 Iași, Romania”. The authors state that the scientific conclusions are unaffected. This correction was approved by the Academic Editor. The original publication has also been updated.
